# m6A regulates breast cancer proliferation and migration through stage-dependent changes in Epithelial to Mesenchymal Transition gene expression

**DOI:** 10.3389/fonc.2023.1268977

**Published:** 2023-11-07

**Authors:** Mohammed G. Dorgham, Brittany A. Elliott, Christopher L. Holley, Kyle D. Mansfield

**Affiliations:** ^1^ Biochemistry and Molecular Biology Department, Brody School of Medicine, East Carolina University, Greenville, NC, United States; ^2^ Department of Medicine, Duke University Medical Center, Durham, NC, United States

**Keywords:** N6-methyladenosine, M6A, RNA modification, breast cancer, Epithelial to Mesenchymal Transition, transformation, mRNA

## Abstract

While many factors have been implicated in breast cancer progression, effective treatments are still lacking. In recent years, it has become clear that posttranscriptional regulation plays a key role in the aberrant gene expression underlying malignancy and metastasis. For example, the mRNA modification N6-methyladenosine (m6A) is involved in numerous post-transcriptional regulation processes and has been implicated in many cancer types, including breast cancer. Despite intense study, even within a single type of cancer, there is little consensus, and often conflicting results, as to the role of m6A, suggesting other factors must influence the process. The goal of this study was to determine if the effects of m6A manipulation on proliferation and migration differed based on the stage of disease progression. Using the MCF10 model of breast cancer, we reduced m6A levels by targeting METTL3, the main cellular m6A RNA methyltransferase. Knocking down Mettl3 at different stages of breast cancer progression indeed shows unique effects at each stage. The early-stage breast cancer line showed a more proliferative phenotype with the knockdown of Mettl3 while the transformed breast cancer line showed a more migratory phenotype. Interestingly, the metastasized breast cancer cell line showed almost no effect on phenotype with the knockdown of Mettl3. Furthermore, transcriptome wide analysis revealed EMT as the probable pathway influencing the phenotypic changes. The results of this study may begin to address the controversy of m6A’s role in cancer and suggest that m6A may have a dynamic role in cancer that depends on the stage of progression.

## 1 Introduction

The National Cancer Institute (NCI) has spent, on average, over 500 million dollars yearly on breast cancer research, and with the addition of private funding the annual average balloons to one billion dollars annually. Despite this, there is still little understanding or effective treatments for advanced disease. The five-year survival rate for breast cancer drops 20 percent between stage 2 and 3 and an additional 50 percent between stage 3 and 4 (1). This deadly progression often involves a process known as Epithelial to Mesenchymal Transition (EMT; 2). Through changes in gene expression, EMT alters the phenotype of the affected cells. In the epithelial stage, proteins that are involved in cell-cell interaction and contact inhibition, such as β-Catenin and E-Cadherin, predominate (3). As the cells progress toward a mesenchymal phenotype, genes involved in proliferation and migration, such as Snail, Vimentin and N-Cadherin, are upregulated (3, 4). Ultimately, this process results in cancer cells that are more migratory, proliferative, and invasive, a deadly trifecta that is difficult to treat.

The N6-methyladenosine (m6A) modification was discovered to be the most common mRNA modification nearly 50 years ago (5). m6A modification of mRNA affects translation efficiency, alternative splicing, mRNA stability, nuclear export, as well as P-body storage (6). This modification occurs via m6A RNA methyltransferases such as Mettl3 and Mettl16 with Mettl3 being the predominant mRNA methyltransferase (7, 8). Mettl3 is part of a complex that contains Mettl14 as well as Wilms tumor associated protein (WTAP). These three proteins form the core of the complex, with Mettl3 being responsible for the catalytic activity via a Rossmann-like fold domain found in other class I methyltransferases. Mettl3 also contains a nuclear localization signal allowing it to enter the nucleus where it utilizes S-adenosylmethionine (SAM) as its substrate, and methylates pre-mRNA. Mettl14 is utilized for substrate binding (mRNA scaffolding) while WTAP helps target the complex to the pre-mRNA (9, 10).

Interestingly, the commonly occurring consensus sequence for Mettl3-Mettl14-WTAP m6A modification (DRACH; underlined A is methylated) is only methylated in 10% of the mRNAs it is found in (11, 12). The m6A modification is also reversible by the m6A demethylases fat mass and obesity related protein (FTO) and alkylation repair homolog 5 (ALKBH5; 8). Furthermore, the m6A modification alone is not enough to induce a regulatory change but is recognized by m6A-specific RNA binding proteins such as the YTH family of proteins (13).

Several studies have begun to link m6A modifications and the progression of cancers through EMT (14–16). However, m6A has been shown to promote tumor progression in breast (16–20) pancreatic (21), gastric (22–24), lung (25, 26) and Acute Myeloid Leukemia (AML (18, 26); cancer, while other studies suggest m6A inhibits tumor progression in breast (15, 18, 27, 28), pancreatic (18), lung (29), cervical (18) and other (28, 30) cancers. This dichotomy leads us to hypothesize that there are additional factors regulating m6A’s specific role in cancer. Our results suggest that the stage of cancer progression may be one of the reasons behind these seemingly conflicting results. As m6A’s role in cancer is being further characterized, these results, as well as future studies must be consolidated to better understand the targets of the modification as well as the mechanisms affecting cancer progression.

## 2 Methods

### 2.1 Cell lines

MCF10A, MCF10AT1 and MCF10CA1h cell lines were obtained from Karmanos Cancer center and maintained on 10 cm dishes (CytoOne, USA Scientific) at 37°C, 5% CO_2_ in DMEM/F12 (Corning) supplemented with 5% Horse serum, 1% Penn/Strep, 0.2% Sodium Chloride, Insulin and Hydrocortisone, 0.07% Cholera toxin and 0.04% Epidermal Growth Factor.

### 2.2 Knockdown of Mettl3

CRISPR/Cas9/sgRNA all-in-one construct co-expressing mCherry (Genecopoeia) was transfected using Lipofectamine 3000 (Thermo Fisher) according to manufacturer’s directions. Cells received either a control sgRNA construct (pCRISPR-CG01) or one targeting exon 1 of Mettl3 (HCP215070-CG12-3-10-a). Two rounds of transfection and Fluorescence Activated Cell Sorting (FACS) were used to isolate mCherry expressing cells which were then diluted to single cell for clonal selection. Western Blotting was used to confirm knockdown of Mettl3.

### 2.3 RNA extraction

Trizol (Life Technologies) was used for all RNA extractions according to the manufacturer’s protocol. RNA for sequencing was further purified and treated with RNase-Free DNase I (Life Technologies) using PureLink RNA Mini Kit (Life Technologies). RNA purity and quantity was determined via NanoDrop 1000 (ThermoFisher Scientific).

### 2.4 Sequencing of PolyA+ RNA

Samples were sequenced by the Brody School of Medicine Integrative Genomics Core. RNA quality and concentration were verified by 4200 TapeStation evaluation (Agilent Technologies) and Qubit Fluorometric Quantitation (Thermo Fisher). Stranded cDNA libraries were prepared using TruSeq Stranded Total RNA prep and ligation with Ribo-Zero Plus kit (Illumina) in accordance with the manufacturer’s protocol. Paired-end sequencing (100 bp × 2) was performed on the NextSeq 2000 system (Illumina).

For differential gene expression analysis, sequence reads were pseudo-aligned to the human hg38 reference transcriptome and transcript abundance quantified by using Kallisto (v.0.46.1). Differential gene expression analyses were achieved by using tximport (v1.20.0) and DESeq2 (v1.26.0) packages in R Studio (Build 351 with R v4.1.1) and further analyzed with GSEA and ShinyGo v0.77 (31). The RNA sequencing data presented in this study are openly available in the GEO database with the accession number GSE237066.

### 2.5 m6A quantification

For UPLC-MS/MS, purified PolyA+ RNA was digested to individual nucleosides and modified nucleosides were quantified as previously described (20).

EpiQuik m6A RNA Methylation Quantification Kit (Colorimetric) was used to quantify relative m6A levels in 400 ng of total RNA according to the manufactures protocol.

### 2.6 Western blotting

Whole cell lysates prepared in whole cell extract buffer (WCEB: 50 mM Tris pH 7.4, 150 mM NaCl, 5 mM EDTA, 0.1% SDS, and complete protease inhibitor (Promega) were quantified with BCA Assay (Promega). Equal protein amounts were electrophoresed on a mini-PROTEAN any KD acrylamide gel (Bio-Rad Laboratories) and transferred to Hybond ECL nitrocellulose (GE Healthcare). The blot was blocked with 5% nonfat dry milk (LabScientific) in Tris buffered saline with 0.1% Tween 20 (TBST) for one hour at room temperature, followed by primary antibody in blocking buffer overnight at 4° C. After washing with TBST, blots were incubated with appropriate secondary HRP conjugated antibody (GE Healthcare), washed again with TBST, detected using Bio-Rad Clarity Western ECL Substrate, and imaged via Invitrogen Ibright FL1500 imaging system. A list of primary antibodies used can be found in **Supplemental Table 1**.

### 2.7 Proliferation

Approximately 2.5x10^5^ cells/well were plated in a 6 well plate. At 24, 48 or 72 hours from plating two wells were lifted with trypsin and counted via trypan blue and hemocytometer in duplicate. Remaining wells were fed with fresh media each day to prevent nutrient/additive deficiency during the time frame of the experiment and to allow for constant proliferation.

### 2.8 Migration

Approximately 2.0x10^6^, 1.5x10^6^, and 1.0x10^6^ cells/well were plated for the MCF10A, MCF10AT1 and MCF10CA1h cell line respectively in a 6 well plate. When the cells reach 95-100% confluency they were serum starved for 4 hours, scratched with a p20 pipette tip, washed with Dulbecco’s Phosphate Buffered Saline (DPBS) and fresh serum free media added. Pictures were taken at 0 and 24 hours and wound healing determined by measuring the change in distance between the cells on both sides of the scratch.

### 2.9 RT-PCR

Reverse transcription was performed on 1 μg of total RNA with the iScript cDNA synthesis kit (Bio-Rad Laboratories). Quantitative real-time PCR was performed using a Roche Lightcycler 96 with Fast Start Essential DNA Green (Roche Diagnostics Corporation) and primers from Integrated DNA Technologies, Inc. Primer efficiency was verified to be over 95% for all primer sets used. Quantification of mRNA was carried out via ΔΔCT analysis using 18S rRNA and the respective control condition for normalization. All real-time PCR primer sets were designed so the products would span at least one intron (>1kb when possible) to prevent detection of the pre-mRNA and/or DNA, and amplification of a single product was confirmed via melting curve analysis. Primer sequences can be found in **Supplemental Table 2**.

## 3 Results

**Figure 1:**
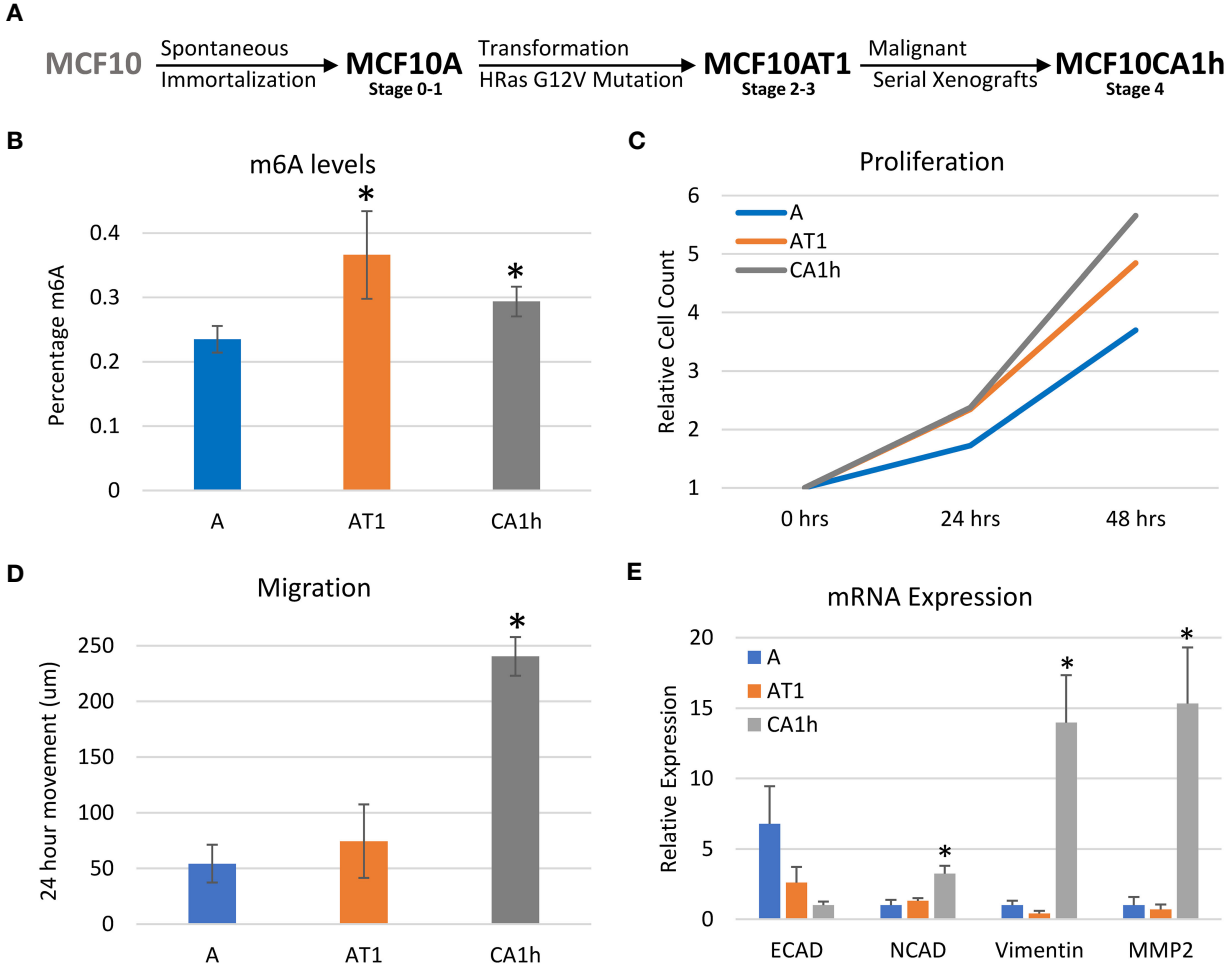
Characterization of the MCF10 model of breast cancer progression. **(A)** Diagram depicting the MCF10 breast cancer model used for this study. **(B)** LC-MS/MS of mRNA nucleosides of the MCF10 breast cancer parental lines showing percent m6A relative to total Adenosine content. **(C)** Proliferation cell count data of parental MCF10 lines. **(D)** Migration/Scratch assay data on parental MCF10 cell lines. **(E)** RNA Expression of major EMT markers across MCF10 parental cell lines. Error bars represent SEM of 3-4 experiments. *p ≤ 0.05 by unpaired Student’s t-test from MCF10A line **(B, E)**, or MCF10A and MCF10AT1 line **(D)**.

This study aimed to characterize the unique role m6A has at differing stages of breast cancer progression utilizing the MCF10 model developed by the Karmanos Cancer Center at Wayne State University. The MCF10 cell lines were originally derived from benign proliferative breast tissue that spontaneously immortalized and are widely used to study cancer progression as well as EMT (32–34). We chose three MCF10 cell lines derived from the same progenitor but representing different stages of progression (**Figure 1A**). The MCF10A’s, which are spontaneously immortalized cells, represent the early stage with a basal-like cell structure. They do not express estrogen receptors and are not tumorigenic (33). The MCF10AT1’s were derived through ectopic expression of mutated HRas (G12V) via stable integration into the MCF10A cell line and are somewhat tumorigenic (33–35). The MCF10CA1h’s were developed from the MCF10AT1 cells via serial mouse xenografts resulting in a highly tumorigenic and metastatic cell line (36, 37). Together, these cell lines represent three distinct stages of breast cancer progression which have all arisen from the same parental cell line.

### 3.1 Characterization of MCF10 model of breast cancer progression

First, the m6A levels of each of the cell lines were determined via LC-MS of polyA selected, rRNA-depleted RNA (20). As shown in **Figure 1B**, the MCF10AT1 cells had the highest percentage m6A-modified adenosines followed by the MCF10CA1h and the MCF10A cells. This initial increase of m6A levels from MCF10A to MCF10AT1 followed by a decrease in m6A levels between the MCF10AT1 and MCF10CA1h suggests that m6A may have a dynamic role in the progression of this breast cancer model.

Next, the cell lines were characterized for the cancer-related phenotypes of proliferation and migration to verify that they modeled breast cancer progression as expected (38). Proliferation was measured by counting the cells every 24 hours over a 72-hour time course while a wound healing assay was used to determine the extent of migration. As expected, there was an increase in proliferation (**Figure 1C**) and migration (**Figure 1D**) as the model progressed from the MCF10A to the MCF10CA1h cells.

Lastly, the state of EMT gene expression was examined to verify that the model also recapitulated known gene expression changes that accompany the epithelial to mesenchymal transition (38). Indeed, there was a decrease in the epithelial marker E-Cadherin and an increase in mesenchymal markers N-Cadherin, Vimentin and MMP2 as the lines progressed from benign to tumorigenic (**Figure 1E**). E-Cadherin was at its highest mRNA expression in the MCF10A line, but its expression was more than halved in the MCF10AT1 cell line and was lowest in the MCF10CA1h cell line which was to be expected as they are the most mesenchymal. There was also a switch from E-Cadherin to N-Cadherin as the model progressed. Interestingly, Vimentin and MMP2 did not show much difference between the MCF10A and MCF10AT1 cell lines but increased dramatically in the MCF10CA1h cell lines.

Overall, the MCF10 breast cancer progression model showed an increase in proliferation and migration between the MCF10A and MCF10AT1, and between the MCF10AT1 and MCF10CA1h cell lines along with a switch from epithelial to mesenchymal markers confirming the model as a valid representation of breast cancer progression.

### 3.2 Effect of knocking down Mettl3 on cellular phenotype

**Figure 2:**
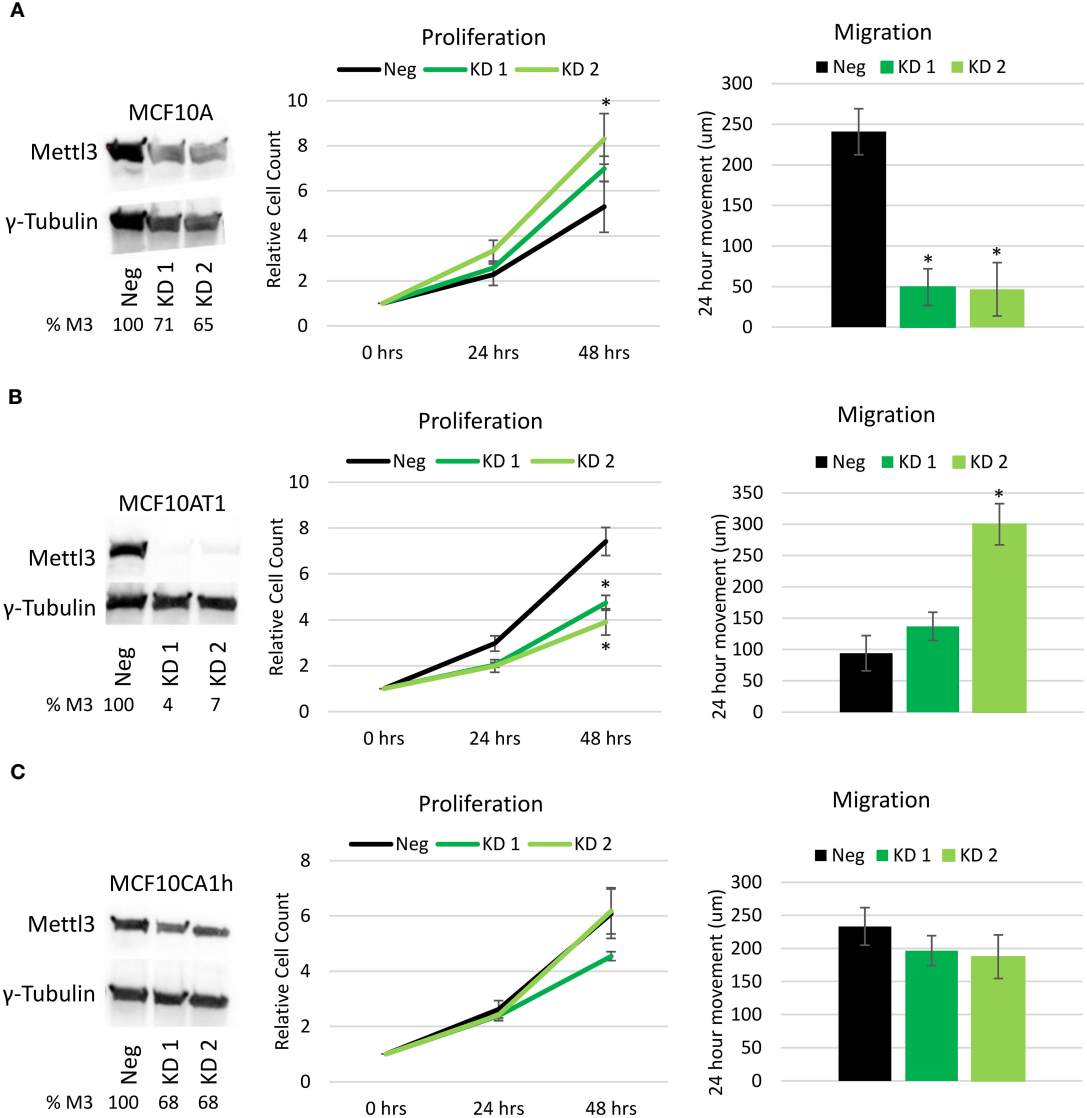
Effect of Mettl3 knockdown on cellular phenotype. Left panels. Western Blots of the **(A)** MCF10A, **(B)** MCF10AT1 and **(C)** MCF10CA1h cell lines with a single negative CRISPR/Cas9 line (Neg) and two different Mettl3 knockdown (KD) cell lines showing Mettl3 protein depletion. Values represent Percent METTL3 protein remaining (% M3) relative to negative line, normalized with gamma-tubulin. (Representative of two experiments.) Middle Panels. Proliferation cell count assays performed over 48 hours on the same cell lines. Right Panels. Migration/Scratch assays performed on the previously mentioned cell lines showing total movement of the cells over 24 hours. Error bars represent SEM of 5 experiments. *p ≤ 0.05 by unpaired Student’s t-test from negative CRISPR cell line.

To investigate the role of m6A in breast cancer progression, Mettl3 was knocked down via transient CRISPR/Cas9 transfection targeting exon 1 coupled with FACS sorting and clonal selection. Knockdown of Mettl3 was confirmed in each cell line via Western blotting and two candidates chosen for each line for use for this study (**Figures 2A–C**). In the MCF10A cell line, knockdown was observed with one candidate having a more apparent knockdown. In the MCF10AT1 cell line there was substantial knockdown in both candidates. TheMCF10CA1h cell line proved to be the most resistant to METTL3 knockdown, as evidenced by only having a moderate level of knockdown in both candidates. Real-time PCR of the lines, while showing lesser of an effect on METTL3 mRNA levels, maintained the relative decrease in METTL3 expression in the knockdown lines (**Supplemental Figure 1**).

To evaluate the effect of m6A decrease on the phenotype of the cells, proliferation and migration assays were run on two CRISPR knockdown candidates and one negative control CRISPR from each of the three cell lines. With the knockdown of Mettl3 in the MCF10A cell line (**Figure 2A**), there was a significant increase in proliferation of one of the knockdown candidates and a significant decrease (almost 80%) in migration in both Mettl3 knockdown lines compared to the negative CRISPR control. Interestingly, the opposite effect was observed in the MCF10AT1 cell lines (**Figure 2B**). With the knockdown of Mettl3, both MCF10AT1 cell lines showed a significant decrease in proliferation and an increase in migration, with one being significant. The MCF10CA1h cell lines showed no significant difference in proliferation or migration (**Figure 2C**). This suggests that m6A via Mettl3 has a dynamic role in the progression of the model and leads to unique changes in phenotype based on the stage of the cancer.

### 3.3 Changes in the transcriptome with the knockdown of Mettl3

**Figure 3:**
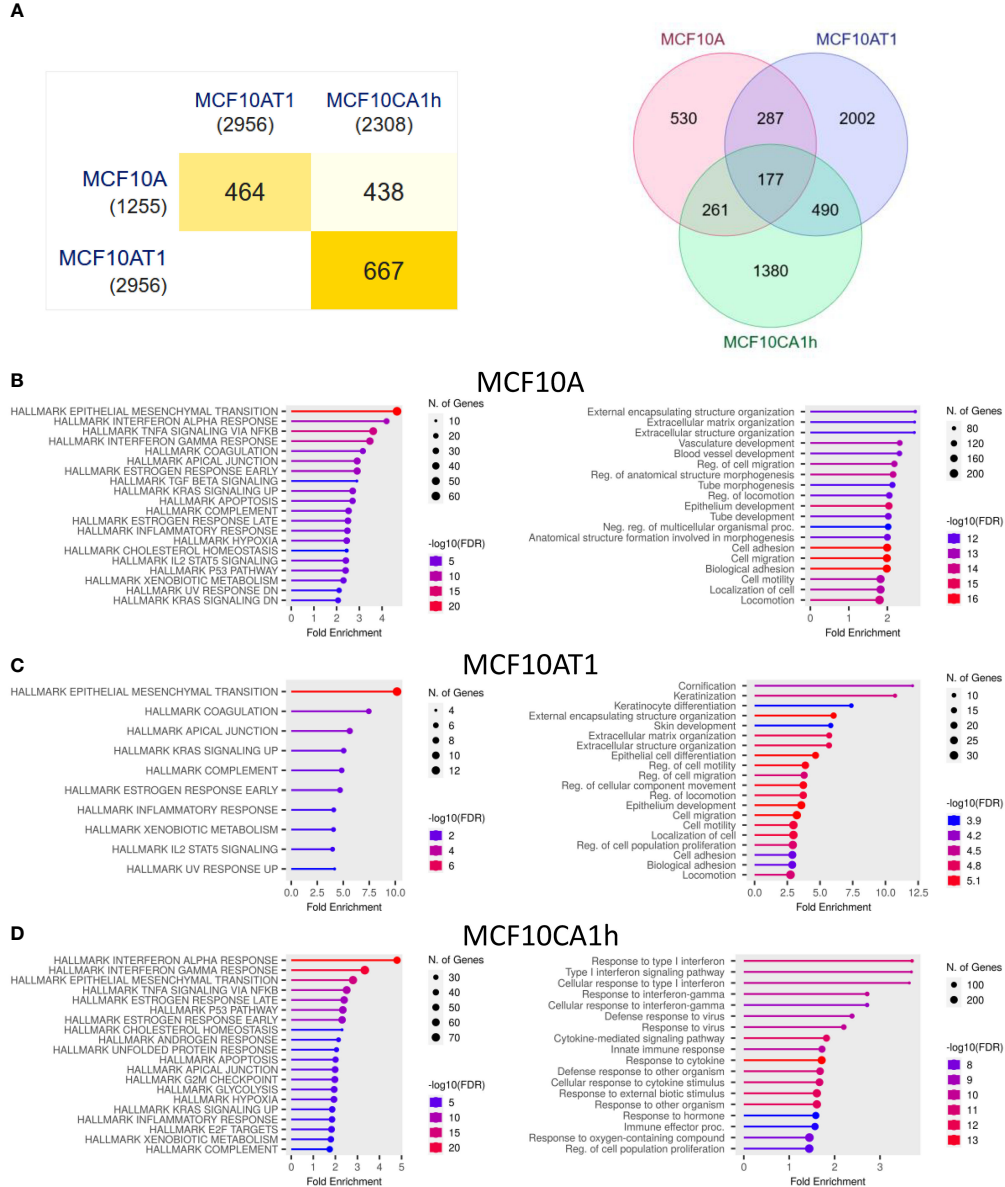
Changes in the transcriptome after Mettl3 knockdown. **(A)** Overlap of all genes significantly different from control in the three cell lines. Left: Pair-wise intersection of the significant genes in each cell line displayed as a minimal triangle matrix. Numbers in (parenthesis) are total number of significant genes for each line. Right: Venn diagram of same data. **(B–D)** Gene set enrichment analysis of the genes significantly different in the Mettl3 CRISPR lines compared to Control CRISPR lines for **(A)** MCF10A, **(B)** MCF10AT1, and **(C)** MCF10CA1h lines respectively. Left: Enrichment charts of the significant gene sets ranked by fold enrichment for the Human MSigDB Hallmarks gene collections. Right: Enrichment charts of the significant gene sets ranked by fold enrichment for the Gene Ontology (GO) Biological Processes gene collections. All analysis was performed with ShinyGo with an FDR cutoff of 0.05.

To determine which genes were most affected by the knockdown of Mettl3, transcriptome wide analysis was performed, and genes identified that differed significantly between the negative CRISPR and one of the knockdown cell lines (**Figure 3A** and **Supplemental Data 1**).

In the three cell lines, 1,255 genes in the MFC10A cell line, 2,956 genes in the MCF10AT1 cell line, and 2,308 genes in the MCF10CA1h cell line showed a significant difference in expression with the knockdown of Mettl3. There was a 464 gene overlap (37.0% and 15.7% of the total for MCF10A and MCF10AT1 respectively) between the MCF10A and MCF10AT1. Similarly, between the MCF10A and MCF10CA1h cell lines 438 genes overlapped (35.0% and 19.0% of the total for MCF10A and MCF10CA1h respectively.) Interestingly, the most overlap of significant gene changes was between the MCF10AT1 and MCF10CA1h with 667 genes overlapping (22.6% and 28.9% of the total for MCF10AT1 and MFC10CA1H respectively.) 177 genes were found to be significantly altered in all three cell lines with the knockdown of Mettl3.

Gene set enrichment analysis was then performed using ShinyGo (31). In the MCF10A cell line, the most significant change was seen in the Hallmark EMT genes (**Figure 3B**). In the GO Biological Processes, genes involved in Extracellular Matrix (ECM) and structure (ECS) organization, regulation of migration, as well as cell adhesion and motility were most significantly affected. The MCF10AT1 cell line also showed the same cluster of Hallmark EMT genes having the most significant change in fold enrichment (**Figure 3C**). Similar to the MCF10A lines, the knockdown of Mettl3 caused a significant change in the Biological Processes of cell motility and migration as well cell adhesion and ECM and ECS organization. In the MCF10CA1h cell line, Hallmark EMT genes were the third most significant cluster of genes. The two most affected gene clusters were Hallmark Interferon Alpha and Gamma response (**Figure 3D**). Interestingly, in the 177 significant genes that overlapped between all three cell lines, the largest cluster within this gene set belonged to Hallmark EMT genes (**Supplemental Figure 2**). This suggests the m6A via Mettl3 plays a significant role in mRNA regulation of EMT messages in breast cancer at all stages.

### 3.4 M6A levels with the knockdown of Mettl3 in breast cancer

**Figure 4:**
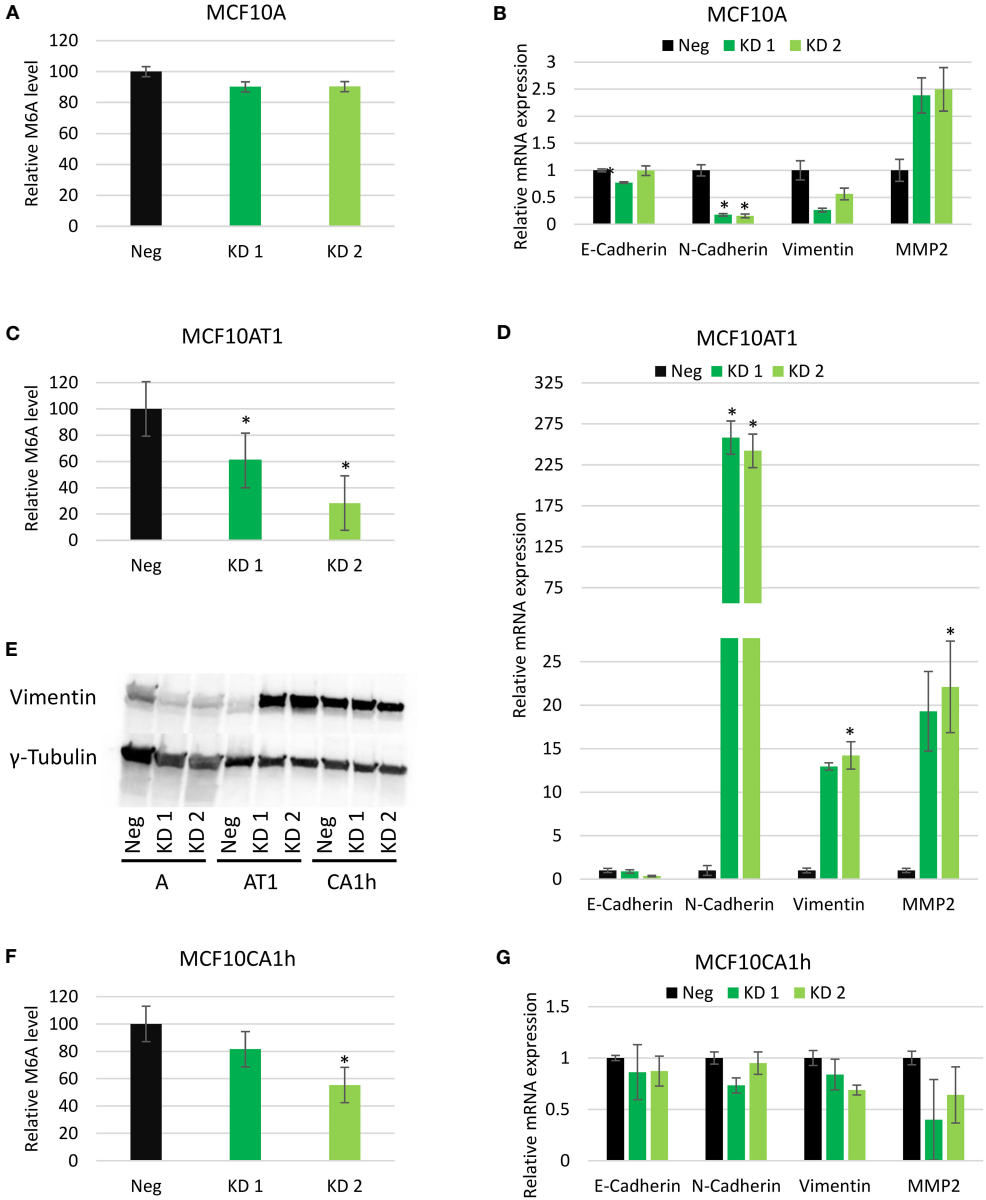
Effect of Mettl3 knockdown on EMT gene expression. **(A, C, F)** Relative m6A quantification from the MCF10A, MCF10AT1, and MCF10CA1h cell lines in the negative control (Neg) and Mettl3 knockdown (KD) lines. Error bars represent SEM of 2 experiments. **(B, D, G)** Relative mRNA expression levels of EMT targets via RT-PCR in same cell lines. Error bars represent SEM of 3 experiments. **(E)** Western Blots of protein lysates from the same MCF10A (A), MCF10AT1 (AT1) and MCF10CA1h (CA1h) cell lines. (Representative of two experiments). *p ≤ 0.05 by unpaired Student’s t-test from negative CRISPR cell line.

Next, m6A levels in total RNA of the cell lines with and without the knockdown of Mettl3 were quantified using a colorimetric, antibody-based quantification kit. With Mettl3 knockdown, a non-significant decrease in m6A was seen in the MCF10A cell line, (**Figure 4A**). This may be due to the overall low basal level of m6A in the MCF10A cell lines (**Figure 1B**) being near the detection limits of the assay. In both the MCF10AT1 and MCF10CA1h lines a significant decrease in m6A levels was observed in the knockdown candidates compared to the negative CRISPR lines (**Figures 4C, F**). Interestingly, even with an unremarkable knockdown of Mettl3 in the MCF10CA1h line (**Figure 2C**) significant drop in m6A levels compared to the negative CRISPR line was still seen.

### 3.5 EMT gene expression

As the EMT pathway was the hallmark most significantly affected by METTL3 knockdown in two of the cell lines (and third in the other), changes in expression of several EMT targets with the knockdown of Mettl3 were examined further by real-time PCR. In the MCF10A cell lines, which typically express high levels of epithelial markers, no change was observed in the mRNA expression of E-Cadherin, but N-Cadherin decreased about six-fold in both knockdowns while Vimentin decreased two to four-fold. Conversely, MMP2 saw a two and half fold increase in both knockdowns of Mettl3 in the MCF10A line (**Figure 4B**). For the MCF10AT1 line, an increase in N-Cadherin, MMP2 and Vimentin was observed in both Mettl3 knockdown lines and a decrease in E-cadherin in one of the lines, consistent with a switch to a mesenchymal phenotype (**Figure 4D**). Vimentin was observed to have thirteen times more expression while MMP2 expression increased about 20-fold with the knockdown of Mettl3. Interestingly, N-Cadherin showed a substantial increase of about 250-fold with the knockdown of Mettl3 compared to the Neg CRISPR in both of the cell lines (**Figure 4D**). Interestingly, similar to the phenotypic results, the Mettl3 knockdown in the MCF10CA1h cell line did not show significant effect on mRNA expression of any EMT targets (**Figure 4G**).

With Vimentin emerging as a regulator of EMT (4) as well as a key player in migration, Vimentin protein levels were determined via Western blot and followed the same trend as the mRNA expression in response to METTL3 knockdown across our three cell lines (**Figure 4E**).

## 4 Discussion

The exact role of m6A in cancer is still emerging. As mentioned previously, there is a plethora of data suggesting that it both promotes and suppresses cancer (15, 17–30, 39), which suggests m6A does not have a static role in cancer progression. Although EMT and m6A are both highly studied topics, the link between the two is still emerging (14, 16, 22, 24, 40). As it is the most abundant mRNA modification in eukaryotes, it is imperative to determine m6A’s dynamic role in cancer progression including how it is involved in EMT.

We have shown that m6A has a more dynamic role in cancer progression than previously thought. In the spontaneously immortalized breast cancer cells (MCF10A), lowering m6A by knocking down Mettl3 caused the cells to become significantly more proliferative. This simultaneously caused the cells to become significantly less migratory as well. Similar results have been shown previously and support our findings (16). It has also been shown that selection between proliferation and migration can vary based on location within the tumor, cell turnover rates, and response to the environment (41). This tradeoff may help explain why METTL3 knockdown would have affected cell proliferation over migration, perhaps due to the abundance of nutrients found in our *in vitro* system.

The transformed MCF10AT1 cells were developed by overexpression of an oncogenic, constitutively active version of HRas. Interestingly, these cells showed an opposite effect in response to the decrease in m6A in that, similar to other breast cancer studies, the proliferation rate significantly decreased, while the migration rate significantly increased (17, 27). This again supports the idea of a tradeoff in that the cells are committing to proliferation or migration. This also clearly shows that within a well-defined model, a small genetic change can not only transform the cells, but also results in utilization of m6A in a different manner. Thus, in the presence of oncogenic G12V HRas, a decrease in m6A brings about an opposite phenotypical change in migration and proliferation as well as a down-regulation of epithelial markers such as E-Cadherin and an upregulation of Mesenchymal markers such as N-Cadherin, MMP2 and Vimentin. Interestingly, we previously saw a similar effect of G12V HRas altering the cellular response to changing m6A levels using a different model of breast cancer progression (20).

In the malignant cell line MCF10CA1h, presumably large genomic changes have occurred during the serial mouse xenografts (33, 36, 37). This cell line is the most proliferative and migratory in this breast cancer model and represents advanced stages of disease. Interestingly, despite our best efforts, it also had the least amount of knockdown of Mettl3 compared to the MCF10A and MCF10AT1 cell lines. While it is possible that this cell line is naturally resistant to CRISPR/Cas9 modification, it is more likely that the cells have become reliant on METTL3 and therefore resistant to its deletion. This potential resistance, as well as the highly proliferative characteristic of these cells, may be the reason for such little change in Mettl3 and no significant effect on proliferation or migration. In addition to possibly not being a large enough knockdown to observe an effect (a theory made less likely by the fact that we did see a m6A decrease and gene expression changes) this resistance to change may be due to the malignant cells being at the upper limit of proliferation and migration rate. If a better knockdown showed a more dramatic result, this could be further progression of the cancer from EMT to Mesenchymal to Epithelial Transition (MET). MET has been shown to occur after metastasized cells have colonized other organs and tissues (38).

We also investigated global changes in the transcriptome resulting from Mettl3 knock down in our breast cancer model to identify pathways that may be regulating the phenotypic changes observed. Interestingly, the cluster of genes that showed the most significant change in fold enrichment was EMT in both the MCF10A and MCF10AT1 cell lines. Consistent with the phenotypic changes, in general EMT genes went down in MCF10A and up in the MCF10AT1 cell lines (**Supplemental Data File 1**). The MCF10CA1h cell line had the most unique gene processes affected with the top five gene processes affected all being related to type I interferon response and signaling. As mentioned before, if the metastasized MCF10CA1h have reached the upper limit of migration and proliferation, then this could answer why we did not see an effect on the phenotypes studied. Furthermore, as m6A appears to have a unique role at different stages of cancer progression, it could affect a much broader range of gene process regulation than previously thought.

Finally, we wanted to verify the changes in expression of several EMT targets with the knockdown of Mettl3 as we did see widespread changes in many EMT pathway genes in all three cell lines (**Supplemental Figure 3**). We observed that Vimentin, and N-Cadherin to lesser extent, was decreased in our knockdown of Mettl3 in our MCF10A cell line. This coincides with our migration data that showed that knocking out Mettl3 decreases migration. Inversely, in the MCF10AT1 we showed an increase in Vimentin, N-Cadherin as well as MMP2 in our knockdown that also showed a significant increase in migration. We saw no significant changes on mRNA expression of our elected EMT targets in the MCF10CA1h line with the knockdown of Mettl3. Since we observed data that both Vimentin and N-Cadherin mRNA expression coincided with the trends seen in our migration data, we elected to run protein expression on Vimentin and that data gave more credence to the idea that Vimentin is the driving force for the changes in migration. This matches the literature that Vimentin has a key role in EMT (4, 42).

We have yet to determine the genes involved in the changes in proliferation in our MCF10A and MCF10AT1 cell lines. Since E-Cadherin is involved in both proliferation and contact inhibition we chose that gene for study but did not observe any substantial evidence that it alone could drive the changes in proliferation. Another likely candidate would be the CDK family of genes as they showed significant changes in mRNA expression in our transcriptome wide analysis (**Supplemental Data File 1**) and have been shown to be involved in cell-cycle progression (43).

Overall, this study has shown that m6A has a unique and dynamic role in EMT progression based the stage of the cancer. Our data also showed that the gene processes affected have some similarities across some of the stages but that there are very unique gene processes regulated by m6A for each stage of breast cancer progression. Lastly, Vimentin showed a significant change in both mRNA and protein expression that followed the same trend of change in migration in both the MCF10A and MCF10AT1 cell lines and to a lesser extent N-Cadherin mRNA expression as well. Thus, while this study does not answer the current conundrum in the field of whether m6A promotes or suppresses cancer, it does suggest that perhaps stage of progression should be a consideration when interpreting those results.

## Data availability statement

The datasets presented in this study can be found in online repositories. The names of the repository/repositories and accession number(s) can be found in the article/**Supplementary Material**.

## Ethics statement

Ethical approval was not required for the studies on humans in accordance with the local legislation and institutional requirements because only commercially available established cell lines were used.

## Author contributions

MD: Conceptualization, Data curation, Formal Analysis, Investigation, Methodology, Writing – original draft, Writing – review & editing. BE: Data curation, Investigation, Writing – review & editing. CH: Data curation, Funding acquisition, Supervision, Writing – review & editing. KM: Conceptualization, Data curation, Formal Analysis, Funding acquisition, Project administration, Supervision, Writing – review & editing.
