# Supplementary material for: m6A regulates breast cancer proliferation and migration through stage-dependent changes in Epithelial to Mesenchymal Transition gene expression
Source: Front Oncol. 2023 Nov 7;13:1268977. doi: 10.3389/fonc.2023.1268977 (PMC10661887; doi:10.3389/fonc.2023.1268977)
Supplement: Supplementary file 2 [file Image_1.pdf]

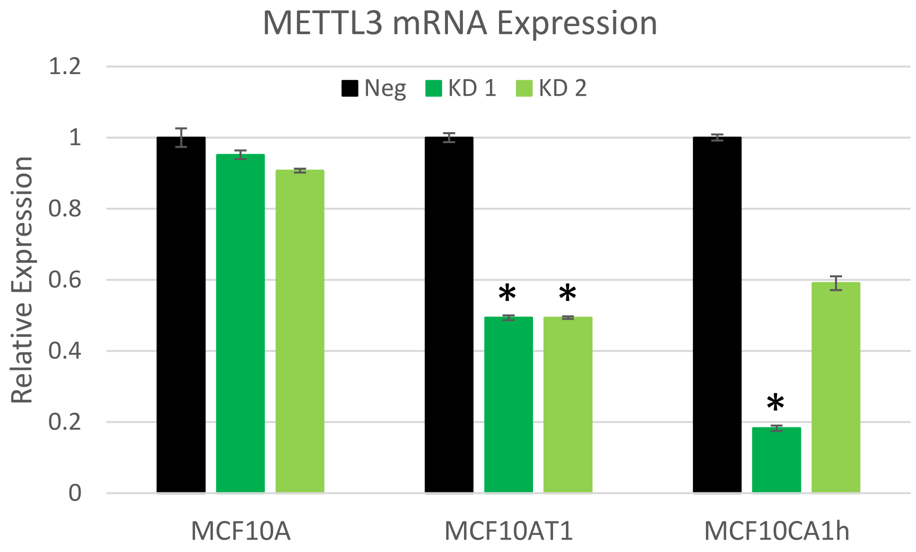

Supplemental Figure 1: METTL3 mRNA Expression of cell lines. Relative METTL3 mRNA quantification from the MCF10A, MCF10AT1, and MCF10CA1h cell lines in the negative control (Neg) and Mettl3 knockdown (KD) lines. Error bars represent SEM of 3 experiments. *$p \leq 0.05$ by unpaired Student's t-test from negative CRISPR cell line.
