# Supplementary material for: m6A regulates breast cancer proliferation and migration through stage-dependent changes in Epithelial to Mesenchymal Transition gene expression
Source: Front Oncol. 2023 Nov 7;13:1268977. doi: 10.3389/fonc.2023.1268977 (PMC10661887; doi:10.3389/fonc.2023.1268977)
Supplement: Supplementary file 3 [file Image_2.pdf]

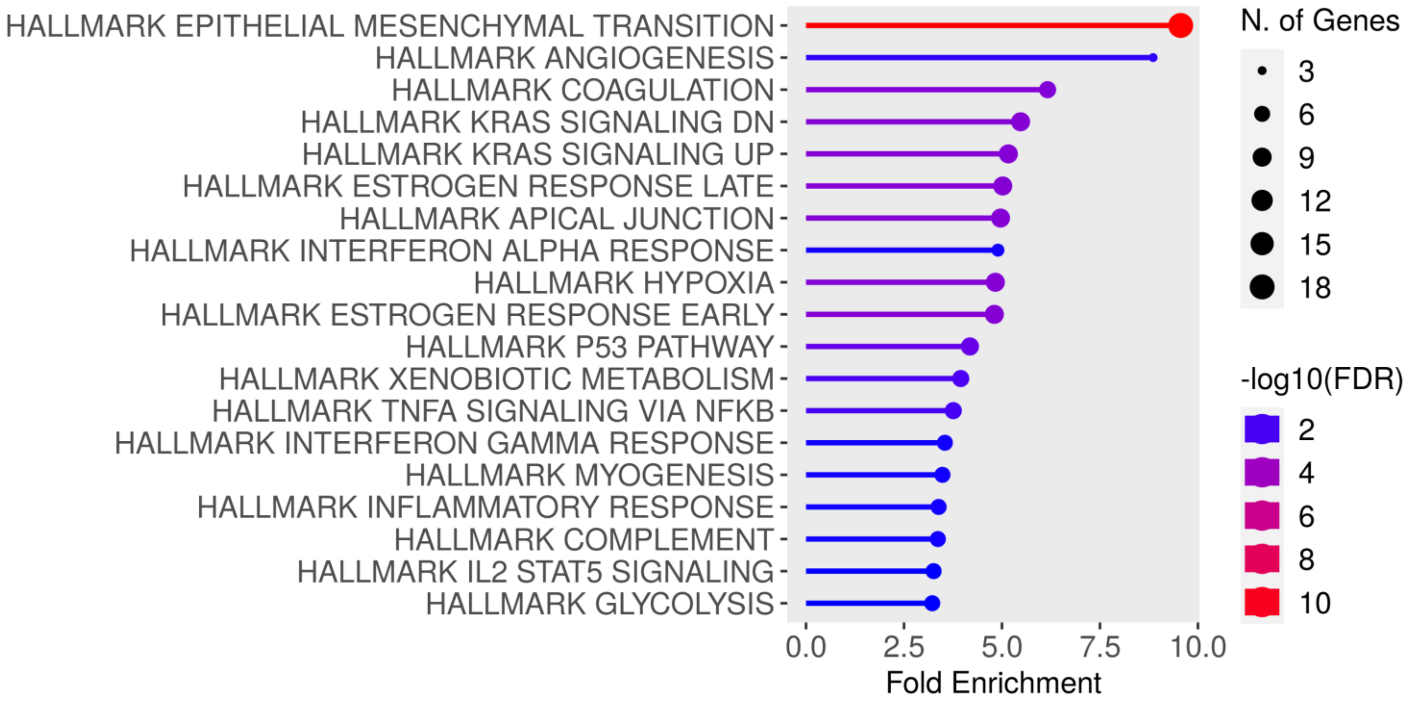

Supplemental Figure 2: Hallmark enrichment of significant gene overlap between lines. Gene set enrichment analysis of the 177 genes significantly different between the Mettl3 CRISPR lines compared to Control CRISPR lines in all three MCF10 cell lines. Shown above is the enrichment chart of the significant gene sets ranked by fold enrichment for the Human MSigDB Hallmarks gene collections. All analysis was performed with ShinyGo with an FDR cutoff of 0.05.
