# Supplementary material for: m6A regulates breast cancer proliferation and migration through stage-dependent changes in Epithelial to Mesenchymal Transition gene expression
Source: Front Oncol. 2023 Nov 7;13:1268977. doi: 10.3389/fonc.2023.1268977 (PMC10661887; doi:10.3389/fonc.2023.1268977)
Supplement: Supplementary file 5 [file Table_1.pdf]

Supplementary Tables 1 and 2

m6A Regulates Breast Cancer Proliferation and Migration through Stage-dependent Changes in Epithelial to Mesenchymal Transition gene expression

Mohammed G. Dorgham, Brittany A. Elliott, Christopher L. Holley, and Kyle D. Mansfield*

*** Correspondence:** Kyle D. Mansfield: mansfieldk@ecu.edu

Supplemental Table 1: Antibodies used in this study

**Product**	**Vendor**	**Catalog #**	**Dilution**
Anti-METTL3	Bethyl Laboratories	A301-567A	1:1000 (WB)
Anti-Gamma Tubulin	ThermoFisher Scientific	MA1-850	1:1000 (WB)
Anti-Vimentin (V9)	Santa Cruz Biotechnoloogy, INC.	sc-6260	1:1000 (WB)
Anti-METTL3 Polyclonal	Protein-Tech	15073-1-AP	1:1000 (WB)

Supplemental Table 2: Real-time PCR primers used in this study

Gene	Forward (5'-3')	Reverse (5'-3')
18S	CTGAGAAACGGCTACCACATC	GCCTCGAAAGAGTCCTGTATT
Mettl3	AGCCTTCTGAACCAACAGTCC	CCGACCTCGAGAGCGAAAT
E-Cadherin	AGAAACAGGATGGCTGAAGG	GCGTGAGAGAAGAGAGTGTATG
N-Cadherin	GACAGTTCCTGAGGGATCAAA	CGATTCTGTACCTCAACATCCC
MMP2	ATGCCGCCTTTAACTGGAG	GGAAAGCCAGGATCCATTTT
Vimentin	CTTCAGAGAGAGGAAGCCGA	ATTCCACTTTGCGTTCAAGG
