# Supplementary material for: m6A regulates breast cancer proliferation and migration through stage-dependent changes in Epithelial to Mesenchymal Transition gene expression
Source: Front Oncol. 2023 Nov 7;13:1268977. doi: 10.3389/fonc.2023.1268977 (PMC10661887; doi:10.3389/fonc.2023.1268977)
Supplement: Supplementary file 4 [file Image_3.pdf]

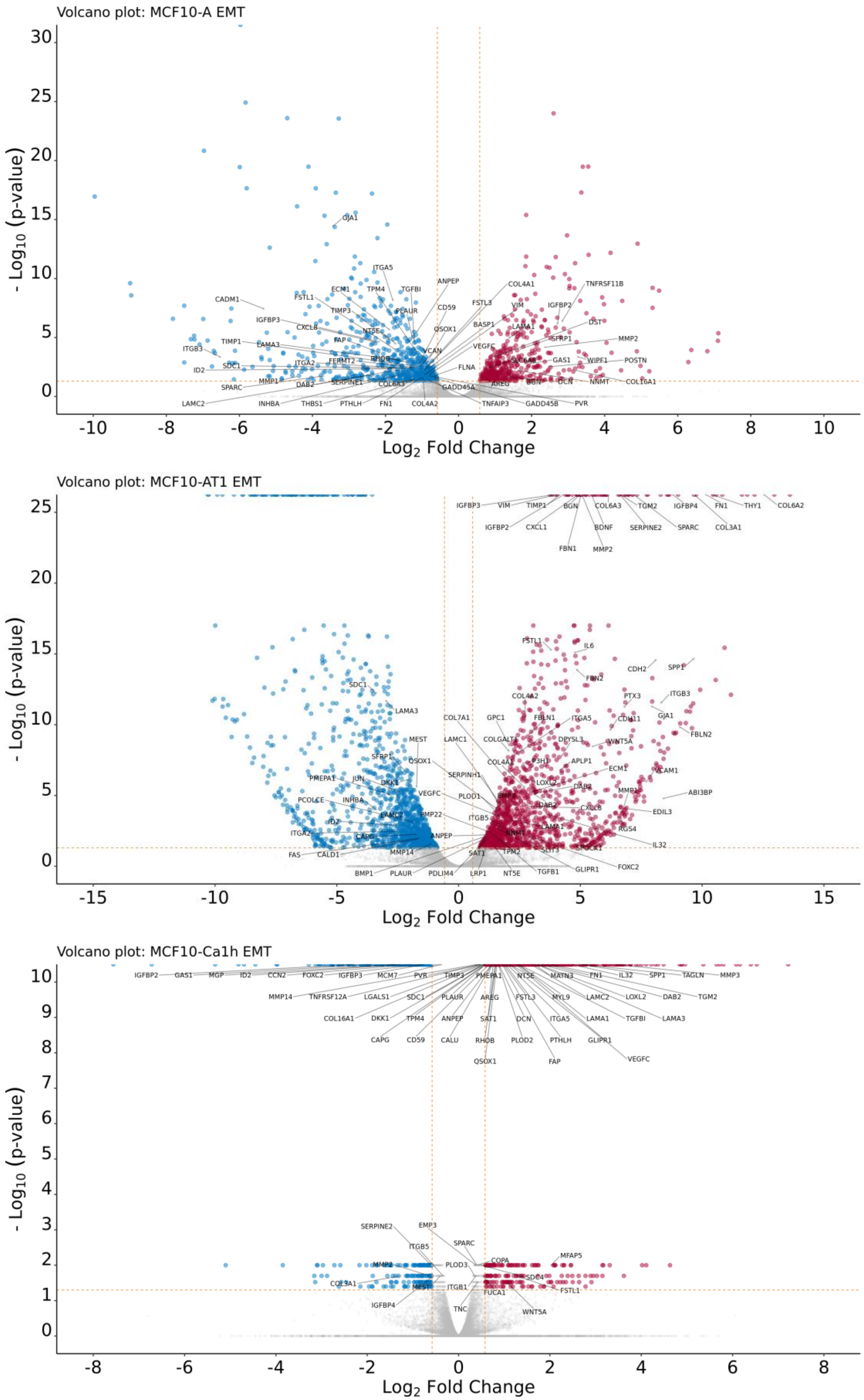

Supplemental Figure 3: Volcano Plots showing distribution of significant EMT genes
Volcano plots of the three MCF10 cell lines with all EMT genes significantly different (Padj<0.05) between WT and M3 knockdown indicated. Plots generated by ggVolcanoR (https://ggvolcanor.erc.monash.edu/)
